# Relation between Mother’s *Taekyo*, Prenatal and Postpartum Depression, and Infant’s Temperament and Colic: A Longitudinal Prospective Approach

**DOI:** 10.3390/ijerph17207691

**Published:** 2020-10-21

**Authors:** Kyung-Sook Bang, Insook Lee, Sungjae Kim, Yunjeong Yi, Iksoo Huh, Sang-Youn Jang, Dasom Kim, Sujin Lee

**Affiliations:** 1 The Research Institute of Nursing Science, College of Nursing, Seoul National University, Seoul 03080, Korea; ksbang@snu.ac.kr (K.-S.B.); lisook@snu.ac.kr (I.L.); sungjae@snu.ac.kr (S.K.); huhixoo1@snu.ac.kr (I.H.); 2 Department of Nursing, Kyung-In Women’s University, Incheon 21041, Korea; yinyis@hanmail.net; 3 College of Nursing, Seoul National University, Seoul 03080, Korea; dudurdaram@naver.com (D.K.); gusangtree@naver.com (S.L.)

**Keywords:** maternal–fetal relations, *taekyo*, depression, infant, temperament, colic

## Abstract

This longitudinal cohort correlational study aimed to confirm the relation among *taekyo* or traditional prenatal practice, prenatal depression, postpartum depression, maternal–fetal interaction, and infant temperament and colic using a prospective design. We recruited 212 women 16–20 weeks pregnant from July 2017 to September 2018; they were followed up until six months postpartum. Data from 97 participants were used in the final analysis. We used the Edinburgh Postnatal Depression Scale, Cranley’s Maternal–Fetal Attachment Scale, and What My Baby Is Like as measurement tools. We observed a significant correlation between prenatal maternal depression in the first to third trimesters and 6–8 weeks and six months postpartum. In addition, infant temperament at six months old showed a significant negative correlation with prenatal and postpartum depression: the higher the prenatal and postpartum depression level, the more difficult the infant’s temperament. *Taekyo* practice was significantly related to maternal–fetal attachment (*r* = 0.45−0.68, *p* < 0.001). Difficult infants showed more colic episodes than any other type of infant (χ^2^ = 18.18, *p* < 0.001). Prenatal and postnatal maternal depression affected infants’ temperament and colic episodes. The management of mothers’ mental health before and after pregnancy is important for infants’ and mothers’ health.

## 1. Introduction

The mother, who is the closest to the baby, is the most important part of the health and development of the infant starting at pregnancy. Pregnancy is a time of transition in which women begin their role as mothers and is an important part of life. At the same time, pregnant women experience many psychological changes, such as anxiety and stress, owing to their new roles and responsibilities as a mother, as well as physical changes [1]. Research has reported that a pregnant woman’s physical and psychological changes cause fatigue and anxiety, and extreme fatigue and anxiety can lead to preeclampsia, preterm birth and prolonged labor [2,3,4]. It is also reported that pregnant women’s stress and anxiety increase the risk of preterm birth, congenital anomaly, intrauterine growth retardation (IUGR), and low birth weight [5,6]. Pregnant women’s anxiety has been reported to significantly correlate with prenatal depression [7] and is a strong predictor of maternal depression [8].

The high prevalence of depression among pregnant women and mothers is a global phenomenon [9,10]. The prevalence of depression in pregnant women has been reported at 5–20%, and that in mothers, at 15–20% [9,10,11]. If a pregnant woman experiences depression, the fetus can be exposed to maternal stress hormones [12,13]. This can affect the fetal stress response system, resulting in vulnerability to anxiety and stress [13]. In addition, the infant of a depressed mother has problems with sleep and has a more difficult temperament [14,15], which can lead to increased maternal depression [16]. Studies have also found that depression in pregnant women and mothers affects the health and development of the fetus and baby [17,18]. Depression in pregnant women affects fetal brain development through physiological mechanisms, and has been reported to be associated with children’s later attention deficit hyperactivity disorder [19]; it also weakens the immune function in infants and young children, thereby increasing the incidence of colic, atopy, and asthma [20].

Meanwhile, in Korea, practicing prenatal care for the fetus is called *taekyo* [21]. *Taekyo*, traditionally regarded as important in East Asia, including Korea [22], refers to the practices for pregnant women: to think positively and speak and act carefully from the early stages of pregnancy until childbirth to have a healthy baby and to have a good influence on the fetus [23]. It is a unique practice according to cultural characteristics and also varies according to changes in the times [24,25,26]. Pregnant women experience emotional exchange with the fetus through various prenatal activities, which leads to the formation of mother–fetus attachment. Maternal–fetal attachment (MFA) is an emotional bond that a pregnant woman has with the fetus; it does not occur spontaneously with the birth of the baby but begins with and continues to develop during pregnancy [27,28,29].

According to the latest data, the average fertility rate of women in member states of the Organization for Economic Cooperation and Development in 2017 averaged 1.65, whereas the total fertility rate in Korea was 0.92 in 2019 [30]. Research related to pregnancy, childbirth, and *taekyo* will be of great significance in understanding social perceptions about MFA, maternal mental health, and the health of infants.

Prenatal depression is defined as a depression that begins during pregnancy [31]. Pregnant women who experience depression during pregnancy become negatively aware of their relationship with the fetus, which also affects the development of the fetus’ growth, which could, in turn, increase the risk of giving birth to low birth weight infants [32,33]. The recent systematic review results have found a significant negative correlation between prenatal depression and maternal–fetal attachment in other cultures [34].

Prenatal depression is considered a strong risk factor for postpartum depression [9,35,36]. Postpartum depression is a depression that begins within six weeks of childbirth and occurs in the first year after childbirth [37,38]. Postpartum depression has multiple symptoms, such as depressed mood, decreased appetite, fatigue, feelings of guilt, insomnia, and suicidal ideation [37]. If maternal women are not emotionally adapted, it negatively affects the pregnant woman’s mental health and maternal attachment with the fetuses and infants [39,40]. Maternal prenatal and postpartum depression are related to unstable attachment, impaired cognitive, behavioral, and emotional development [41,42,43]. Therefore, it is important to manage maternal health care at an early stage.

However, local studies have not used an integrated approach to investigate the relation among the practice of *taekyo*, MFA, maternal mental health, and infant’s health. Moreover, some studies have been conducted on postpartum depression in pregnant women and its effects on the baby, but research on the relation between prenatal depression and postpartum depression, and how depression affects the growth and health of infants remains limited. Particularly, prospective longitudinal studies on this topic are difficult to find.

Therefore, we aimed to identify the maternal depression status and clarify the relationship between maternal depression and the infant’s health by the prospective longitudinal method. This study will contribute to improving understanding of the importance of prenatal care for mothers and infants. The purpose of this study was to confirm the relation between *taekyo* practice, prenatal depression, postpartum depression, MFA, and infant temperament and colic in a prospective manner. Specific research hypotheses of this study were as follows:

1. Maternal prenatal depression will be correlated with postpartum depression.
2. Maternal *taekyo* practice will be correlated with maternal–fetal attachment and prenatal–postpartum depression.
3. Maternal prenatal-postpartum depression will be correlated with the infant’s temperament and colic.

## 2. Materials and Methods

### 2.1. Study Design

We used a longitudinal cohort correlational design.

### 2.2. Study Sample

Participants were pregnant women between 16 and 20 weeks of pregnancy at the time of inclusion. For correlation analysis by Cohen [44], the required sample size was 84 when calculated based on an effect size of 0.30, a significance level of 0.05, and statistical power of 0.80. In this study, considering a 50% dropout rate, as long-term follow-up surveys were conducted from 16 weeks of pregnancy to six months postpartum, 168 participants were needed. We recruited 212 participants, and the final sample consisted of 97 participants. The dropout rate was 54%, but our study met the sample size required. We tried to prevent dropouts by giving gift certificates for each survey and giving baby items after childbirth. Nonetheless, dropouts occurred in the follow-up survey that lasted a year.

Nulty [45] states that the response rate above 70% is desirable depending on the number of participants, but at the same time, the previous study shows that the paper-based response rate is an average of 56%, and the online response rate is an average of 33% [45]. In this study, five surveys were conducted, both written and online. The first and third surveys were done in writing at the time of the pregnant woman’s visit to the hospital, while the second, fourth and fifth surveys were done online whilst not visiting the hospital. It was difficult to find previous studies that used the same research method as our study, but a domestic longitudinal study of subjects and participants similar to this study confirmed that the dropout rate was approximately 50% [46]. Therefore, our longitudinal study also considered a dropout rate of 50%.

A total of 97 pregnant women were recruited from July 2017 to September 2018 and were followed up until six months postpartum. Finally, data from 97 participants were used in the final analysis. The participants were recruited at a maternity hospital in Jeonju, South Korea. A research assistant with a registered nurse license checked the medical history and hospital records of the participants who met the inclusion criteria: (1) agreeing to participate in the study; (2) having no underlying diseases (hypertension, diabetes, heart disease, and thyroid disease); and (3) being pregnant with no experience of depression and anxiety. The exclusion criteria were as follows: (1) high-risk pregnancy (e.g., placenta previa, preeclampsia, gestational diabetes, and high spontaneous abortion risk); (2) preterm birth (childbirth before 37 weeks of pregnancy), stillbirth, and abortion.

### 2.3. Instruments

#### 2.3.1. Demographic Data

We collected the following general characteristics of the participants: obstetric history, morbidity (past history, family history), smoking experience, drinking, physical activity, and vital signs, including blood pressure and heart rate.

#### 2.3.2. *Taekyo* Practice

*Taekyo* practice was measured using Ahn’s tool [47] which we modified and supplemented to measure the sub-domains of *taekyo* practice. The modified tool consisted of 11 items, with each item rated on a five-point scale. Higher scores indicated a higher degree *taekyo* practice.

#### 2.3.3. Depression

Prenatal depression was measured using the Edinburg Postnatal Depression Scale (EPDS), which was developed by Cox, Holden, and Sagovsky [48] and adapted by Yeo [49]. EPDS is one of the most widely used tools for assessing prenatal and postpartum depression. The scale contains ten items, each scored from zero to three (0 = no depression; 3 = high depression) on a Likert scale; the total score ranges from zero to 30, with scores above 13 indicating depression. The Cronbach’s alpha coefficient was 0.76 in Yeo [49] and 0.84 in our study.

#### 2.3.4. Maternal–Fetal Attachment

We used Cranley’s Maternal–Fetal Attachment Scale modified by Lee [50] for assessing MFA. This scale consists of 16 items in five domains, namely, “Role taking,” “Differentiation of self from fetus,” “Interaction with fetus,” “Attributing characteristics to fetus,” and “Giving of self.” The items are scored on a five-point Likert scale, in which 1 = Definitely Yes and 5 = Definitely No. The total score ranges from 16 to 80. Higher scores indicate greater attachment. The internal consistency reliability of the scale was confirmed by its original designer (α = 0.85) [27,51]. In our study, Cronbach’s alpha was 0.86.

#### 2.3.5. Infant Temperament

Infant temperament was measured on a nine-point scale of seven questions evaluating warmth and continuity in What My Baby Is Like, developed by Pridham, Chang, and Chiu [52]. The higher the score, the easier is the baby to soothe, indicating a baby with a regular and consistent positive temperament. In Korea, the validity and reliability of the scale were confirmed in Bang’s study [53], and Cronbach’s alpha was 0.81. In our study, Cronbach’s alpha was 0.87.

#### 2.3.6. Infant Colic

Infant colic was scored 1 point for “Yes” and 0 for “No” depending on whether or not it occurred during the postpartum period.

### 2.4. Data Collection

This study was approved by the Institutional Review Board at Seoul National University (IRB No. 1707/003-004). Data collection was conducted at the obstetrics and gynecology hospital in Jeonju, a research cooperation institution. Data collection was conducted by a nurse as a research assistant. Before beginning data collection, we explained the study’s purpose, methods, and procedures, and the benefits and risks of participation to the participants. The participants were told that they could be discontinued from participating at any time. Those who agreed to participate in this study provided their informed consent.

Data were measured at 16–20, 24–28, and 33–35 weeks of gestation, 6–8 weeks postpartum, and six months postpartum. The first survey (16–20 weeks) measured demographic and obstetric data, maternal depression, and MFA. The second and third surveys (24–28 and 33–35 weeks) measured *taekyo* practice, maternal depression, and MFA. The fourth and fifth surveys (6–8 weeks and 6 months postpartum) measured maternal depression, infant’s temperament, and colic. The first to third surveys were conducted to identify the physical and emotional state of the mother and fetus before childbirth. The fourth to fifth surveys were conducted to determine the longitudinal effects of the mother’s emotion and infant’s health condition after childbirth.

The methods of investigation were carried out through written and online questionnaires. The first and third surveys were conducted as written questionnaires when the participants visited hospitals for prenatal examinations, and the second, fourth, and fifth surveys were conducted as online questionnaires through the contact information collected with the participant’s consent. The methods of the investigation were designed to reduce the dropout rate of participants in the study. The collected data were assured of anonymity and confidentiality.

### 2.5. Data Analyses

Data were analyzed using SPSS Statistics for Windows, version 25 (IBM Corp., Armonk, NY, USA). Descriptive statistics were used to describe the participants’ demographic and obstetric data and included frequency, percentage, mean, and standard deviation. Data normality was examined using skewness and kurtosis, and all the continuous data were found to have normal distribution. Skewness and kurtosis between −2 and +2 were considered as acceptable to prove normal distribution. To perform the bivariate analysis, we used Pearson’s correlation coefficient to determine the relationship among *taekyo* practice, MFA, maternal depression, infant’s temperament, and colic. The Fisher’s exact test was used to determine colic occurrence according to infant temperament. Statistical significance was set at *p* < 0.05.

## 3. Results

### 3.1. Characteristics of Participants and Descriptive Data

Table 1 presents the demographic characteristics of the participants, including smoking, drinking, and exercise status. A total of 97 participants were included in the study. The majority of the participants were aged 24–34 years; the average age was 30.27 ± 3.31 years. More than half of the participants had planned pregnancies (51.5%), and 60.8% had an occupation. Household income was classified based on a median income of 150% (KRW 4,490,000). More than half of the participants (54.7%) belonged to the 150% median income group, whereas 45.3% belonged to the under-150% median income group. Of the participants, 19.6% had a history of smoking, none drank or smoked during pregnancy (100.0%), and 71.1% exercised during pregnancy.

### 3.2. Prenatal and Postpartum Depression Status and High Risk Depression Ratio

Table 2 presents the depression scores by period and high-risk depression ratio (≥13 score). The survey was conducted from 16 to 20 weeks of gestation to six months postpartum. By period, the depression scores were 6.43 ± 3.96 at 16–20 weeks, 5.89 ± 4.18 at 24–28 weeks, and 6.33 ± 3.90 at 33–35 weeks. At postpartum follow-up, the depression score was 7.60 ± 4.78 at 6–8 weeks after childbirth and 6.94 ± 5.03 at six months after childbirth.

Depression scores of ≥13 indicated high-risk depression in our study. Of the participants, 8.2% were at high risk at 16–20 weeks, 5.2% at 24–28 and 33–35 weeks, 13.4% at 6–8 weeks postpartum, and 15.5% at six months postpartum.

### 3.3. Maternal Taekyo Practice, Maternal–Fetal Interaction, Prenatal–Postpartum Depression and Infant Temperament

Table 3 presents a significant correlation between prenatal depression in the first to third trimesters, and postpartum depression (6–8 weeks and six months). Therefore, depression tended to persist from the prenatal period to six months postpartum.

*Taekyo* practice (*p* < 0.001) at 24–28 and 33–35 weeks showed a significant correlation with MFA at that time (*r* = 0.45, *p* < 0.001; *r* = 0.68, *p* < 0.001). We found no significant correlation between *taekyo* practice and prenatal depression. *Taekyo* practice also showed no correlation with depression 6–8 weeks after childbirth, but it was reversely related to depression at six months after childbirth.

In terms of infant temperament, 6–8 weeks of age did not show a significant correlation with prenatal and postpartum depression of the mother because the infant temperament was not clearly revealed. However, the infant temperament at six months of age showed a significant negative correlation with prenatal and postpartum depression. Thus, the higher the prenatal and postpartum depression, the more difficult the infant’s temperament is.

### 3.4. Infant Temperament and Colic

Table 4 presents comparison of colic occurrence according to infant temperament. Of all infants, infants with difficult temperament accounted for 11.3%, infants with moderate temperament, 35.1%, and infants with easy temperament, 53.6%. In comparing the occurrence of infant colic according to temperament, we found that infant colic occurred in 45.5% and 35.3% of infants with difficult and moderate temperament, respectively. Only 3.8% of infants with easy temperament showed colic. We also found a statistically significant difference (χ^2^ = 18.18, *p* < 0.001).

## 4. Discussion

The present study aimed to investigate the relation among *taekyo* practice, prenatal depression, postpartum depression, MFA, infant’s temperament, and colic from the first trimester of pregnancy to six months after childbirth.

In Eastern society, especially in Korea, the importance of *taekyo* has been emphasized since ancient times [54]. In the Korean Folklore Encyclopedia, *taekyo* is defined as a concept that includes thoughts, mindset, and behaviors as “the thoughts or actual actions taken by pregnant women to have a good effect on a pregnant fetus” [55]. From the prenatal period, the emotional state of pregnant women is known to affect the health and development of mothers and babies.

In our study, *taekyo* practice showed a significant correlation with mother–fetus interaction, supporting the results of previous studies [28,29]. *Taekyo* practice recognizes the importance of physical health management and psychological stability for mothers from the prenatal period. However, doing well may also be a burden and responsibility for pregnant women. Indeed, although *taekyo* practice showed a significant correlation with mother-fetus interaction, it did not decrease the mother’s depression. Therefore, rather than emphasizing unilateral and traditional *taekyo* practice, interventions that relieve depression by focusing on the emotional state of the mother may be more important to both mother and baby.

In recent years, prenatal and postpartum depression have received increased interest worldwide, with increasing awareness that depression affects not only the mother’s mental problems but also the baby. A mother’s mental health during pregnancy and the first year postpartum is of the utmost importance to the cognitive, social, and emotional development of the child [18]. However, few longitudinal studies have been conducted on the relation between prenatal and postpartum depression in Korea. We used a longitudinal prospective sample to examine the long-term course of maternal depression and its association with infant temperament and health problems. We longitudinally tracked depression in the first, second, and third trimester, and the in postnatal period up to six months postpartum. These results confirmed the high correlation between depression and postpartum depression at various prenatal time points. Lee and Park [56] also reported that prenatal depression is a factor that predicts postpartum depression. These results indicate that active management of prenatal depression is necessary from the time of pregnancy.

The baby learns the world and builds confidence in human relationships through interaction with the mother; such interaction between the mother and the baby is a crucial foundation in life. In our study, MFA did not show a direct correlation with prenatal depression but showed a significant correlation with *taekyo* practice. These results support previous findings [57] that prenatal MFA is related to postnatal depression and that MFA is related to *taekyo* practice [28]. Therefore, *taekyo* ought to focus on the practicing mother recognizing the fetus as a lovely being and expressing mutual affection as they progress toward the end stage of pregnancy, rather than focus on the responsibility to be fulfilled for the baby.

We also examined how the mother’s prenatal and postpartum depression affected the baby. The results of this study revealed that the most significant factors affecting the baby’s temperament were the prenatal and postpartum depression of the mother, compared with prenatal or maternal interactions. Our findings support previous results suggesting that anxiety and depression in a mother’s pregnancy are associated with babies with less expressive of joy, a lack of positive emotions, and an irregular, fearful, and demanding temperament [58]. A study in Switzerland reported the significant association between maternal health and mood problems in the immediate postpartum period and midwife-reported crying problems in infants. When the mother was depressed, the occurrence of crying problems in the baby had an adjusted odds ratio 4.02 compared with the case where she was not depressed, which was the most influential factor. Newborns exposed to maternal depression show more dysregulated behavior, such as disturbed/disorganized sleep and difficult temperament [14,15], which can reciprocally further exacerbate maternal depression [16].

In addition, Hanington, Ramchandani, and Stein [59] showed that postpartum depression affects infants’ temperament. Bang [46] reported that infants in a postpartum depression maternal group show significantly lower amenability and persistence compared with infants in a general maternal group, and the former also have a more difficult temperament than the latter. In our study, the incidence of infant colic was high in cases of difficult infant temperament and low in cases of moderate or mild infant temperament. These findings are in accordance with previous reports [60]. Indeed, Neu and Robinson [61] confirmed that infants with colic tend to have more difficulty in emotional regulation. Thus, prenatal, and postnatal depression, infant temperament, and infant colic are related to one another. Therefore, a strategy for managing depression from the early stages of pregnancy is considered essential, as it can be directly linked to maternal and infant health.

This study’s results were summarized according to effect sizes, as defined by Cohen [44]. The effect size is defined as the degree of the effects of a predictor on other variables [62] and is used to evaluate statistical significance [63]. According to Cohen [44], correlation studies’ effect size is used to the *r*-value of 0.10, 0.30, and 0.50 for small, medium, and large effects, respectively. In this study, it is considered that there is a large effect size between *taekyo* practice and MFA (*r* = 0.45–0.68), and a medium effect size between maternal prenatal–postpartum depression and six-month infant temperament (*r* = −0.26, −0.30).

In a previous study, Jang and Bang [28] reported the association between *taekyo* practice and MFA (*r* = 0.65), and Yu and Kim [29] found evidence for a reliable correlation between *taekyo* practice and MFA (*r* = 0.71, *p* < 0.001). The strong effect size between *taekyo* practice and MFA in the prior study was similar to this study’s results. Moreover, the effects of maternal depression and infant temperament were also reported by several research groups. Rouse and Goodman [64] found that prenatal depression was associated with infant negative effects after controlling for postpartum symptoms. Kwon et al. [65] reported the correlation between postpartum depression and infant temperament (*r* = 0.18, *p* < 0.001). In addition, Bang [46] found that mothers in the postpartum depression group’s babies had more difficult temperaments than normal maternal groups (*p* < 0.001). Along with previous studies, this study found a significant effect between *taekyo* practice and maternal–fetal attachment, and also, maternal depression and six-month infant temperament.

This study has several strengths. We tracked and analyzed longitudinal data from the first to third trimester of pregnancy up to six months after childbirth to produce meaningful results. In Korea, research on the relation between prenatal depression and postpartum depression and how these affect the health of infants is limited. Our study revealed the correlation between prenatal and postpartum depression and is meaningful in that prenatal and postpartum depression are related to the temperament of infants. In addition, the result that *taekyo* practice is related to MFA and postpartum depression at six months suggests the importance of prenatal care.

As for the limitations, first, all participants were recruited from a local area in Korea; therefore, this study’s results are difficult to generalize. To generalize the results of this study, further research needs to be conducted on a nationwide scale. Second, we were not able to recruit large-scale samples because the participants were recruited when the domestic birth rate was declining. Lastly, the dropout rate is above 50% in this study. Future research needs to consider various ways to prevent the dropout of research participants. Given these limitations, future research should be conducted to recruit large-scale samples by expanding the scope of recruitment areas to various regions in Korea. Nonetheless, this study suggests meaningful findings from the data of the recruited participants. This study has contributed to the first longitudinal research in Korea that examines the relation of mother’s *taekyo*, prenatal–postpartum depression, and infant’s temperament and colic.

## 5. Conclusions

In this study, we found a correlation between *taekyo* practice and MFA, and that *taekyo* practice and postpartum depression at six months had a reverse correlation. Thus, MFA can be strengthened, and postpartum depression can be reduced for pregnant women who carry out *taekyo* practice well before childbirth. Additionally, we confirmed that prenatal and postpartum depression were correlated with the infant’s temperament. In other words, prenatal depression can affect postpartum depression and the infant’s temperament. These findings prove the importance of *taekyo* practice and suggest that more active intervention is needed through prenatal depression screening from the early stages of pregnancy. According to the findings, maternal depression is one of the key factors related mothers and infants’ health. This study can provide evidence of the importance of maternal psychological care as well as physical care. Furthermore, the study found that maternal depression is related to fetal attachment and infant temperament. Therefore, the study contributed to supporting the importance of maternal emotional care. In the future study, we suggest a large-scale follow-up study in Korea, which will contribute to the generalization of research findings.

## Figures and Tables

**Table 1:** Demographic characteristics of participants (*N* = 97).

Characteristics	Categories	n (%) or M ± SD
Maternal age (years)	24–34	86 (88.7)
	≥35	11 (11.3)
		30.27 ± 3.31
Planned pregnancy	Yes	50 (51.5)
	No	47 (48.5)
Mother’s occupation	Yes	59 (60.8)
	No	38 (39.2)
Spouse’s occupation	Yes	95 (97.9)
	No	2 (2.1)
Monthly income (KRW 10,000)	<150% median income	53 (54.7)
	≥150% median income	44 (45.3)

**Table 2:** Depression score by period (*N* = 97).

Variables	n	M ± SD	Range	Number of High-Risk Mothers (%)
1. Depression (16–20weeks of gestation)	97	6.43 ± 3.96	0–17	8 (8.2)
2. Depression (24–28weeks of gestation) *	89	5.89 ± 4.18	0–23	5 (5.2)
3. Depression (33–35weeks of gestation) *	96	6.33 ± 3.90	0–19	5 (5.2)
4. Depression (6–8weeks postpartum)	97	7.60 ± 4.78	0–22	13 (13.4)
5. Depression (6 months postpartum)	97	6.94 ± 5.03	0–21	15 (15.5)

* Missing data were excluded.

**Table 3:** Pairwise correlations among *taekyo* practice, maternal–fetal attachment, prenatal–postpartum depression, and infant temperament (*N* = 97).

Variables	1	2	3	4	5	6	7	8	9	10	11	12
	r (*p*)											
1. *Taekyo* practice (24–28 weeks of gestation) ^†^	1											
2. *Taekyo* practice (33–35 weeks of gestation) ^†^	0.72 **	1										
3. Maternal–fetal attachment (16–20 weeks of gestation)	0.46 **	0.58 **	1									
4. Maternal–fetal attachment (24–28 weeks of gestation) ^†^	0.45 **	0.46 **	0.63 **	1								
5. Maternal–fetal attachment (33–35 weeks of gestation) ^†^	0.47 **	0.68 **	0.68 **	0.69 **	1							
6. Depression (16–20 weeks of gestation)	−0.11	−0.17	−0.05	0.08	−0.02	1						
7. Depression (24–28 weeks of gestation) ^†^	−0.14	−0.01	0.02	−0.16	0.02	0.60 **	1					
8. Depression (33–35 weeks of gestation) ^†^	−0.11	−0.13	0.00	−0.05	−0.01	0.67 **	0.69 **	1				
9. Depression (6–8 weeks postpartum)	−0.14	−0.14	0.06	0.01	−0.02	0.40 **	0.51 **	0.51 **	1			
10. Depression (six months postpartum)	−0.22 *	−0.31 **	−0.11	−0.09	−0.27 **	0.53 **	0.48 **	0.45 **	0.50 **	1		
11. Infant’s temperament (6–8 weeks postpartum)	−0.03	−0.04	0.01	0.05	0.01	−0.15	−0.12	−0.14	−0.10	−0.11	1	
12. Infant’s temperament (six months postpartum)	0.18	0.20	0.18	0.11	0.11	−0.16	−0.26 *	−0.20	−0.23 *	−0.30 **	0.17	1

* *p* < 0.05, ** *p* < 0.01, ^†^ Missing data were excluded.

**Table 4:** Comparison of colic occurrence according to infant temperament (*N* = 97).

Variables	Categories	n (%)	Infant Colic (n = 97)		χ^2^	df	*p* *
			Yes	No			
			n (%)	n (%)			
Infant’s temperament	Difficult	11 (11.3)	5 (45.5)	6 (54.5)	18.18	2	<0.001
	Moderate	34 (35.1)	12 (35.3)	22 (64.7)			
	Easy	52 (53.6)	2 (3.8)	50 (96.2)			

* Fisher’s exact test.
